# Rice-derived arabinoxylan fibers are particle size-dependent inducers of trained immunity in a human macrophage-intestinal epithelial cell co-culture model

**DOI:** 10.1016/j.crfs.2023.100666

**Published:** 2023-12-20

**Authors:** Bart G.J. Moerings, Suzanne Abbring, Monic M.M. Tomassen, Henk A. Schols, Renger F. Witkamp, Klaske van Norren, Coen Govers, Jeroen van Bergenhenegouwen, Jurriaan J. Mes

**Affiliations:** a Division of Human Nutrition and Health, Wageningen University & Research, Wageningen, the Netherlands; b Wageningen Food and Biobased Research, Wageningen University & Research, Wageningen, the Netherlands; c Laboratory of Food Chemistry, Wageningen University & Research, Wageningen, the Netherlands; d Cell Biology and Immunology Group, Wageningen University & Research, Wageningen, the Netherlands; e Danone Nutricia Research, Utrecht, the Netherlands

**Keywords:** Arabinoxylan, Co-culture model, Dectin-1, Particle size, Trained immunity

## Abstract

Arabinoxylans have been identified for a wide range of purported health-promoting applications, primarily attributed to its immunomodulatory effects. Previously, we have reported the ability of arabinoxylans to induce non-specific memory in innate immune cells, commonly referred to as “trained innate immunity”. In the present study, we investigated the effect of particle size on innate immune training and resilience in primary human macrophages as well as in a more physiologically relevant macrophage-intestinal epithelial cell co-culture model. We demonstrated that smaller (>45 & < 90 μm) compared to larger (>90 μm) particle size fractions of rice bran-derived arabinoxylan preparations have a higher enhancing effect on training and resilience in both models. Smaller particle size fractions elevated TNF-α production in primary macrophages and enhanced Dectin-1 receptor activation in reporter cell lines compared to larger particles. Responses were arabinoxylan source specific as only the rice-derived arabinoxylans showed these immune-supportive effects. This particle size-dependent induction of trained immunity was confirmed in the established co-culture model. These findings demonstrate the influence of particle size on the immunomodulatory potential of arabinoxylans, provide further insight into the structure-activity relationship, and offer new opportunities to optimize the immune-enhancing effects of these dietary fibers.

## Highlights

• Rice-derived arabinoxylans induce particle size-dependent trained immunity.
• Smaller particle size fractions exhibited heightened Dectin-1 receptor activation.
• A co-culture model demonstrated training and resilience of small rice bran-1 fractions.

## Graphical abstract

**Figure:**
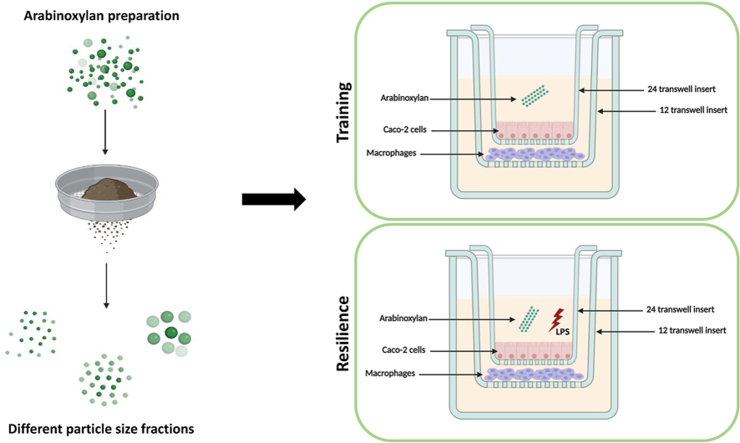

## 1 Introduction

Dietary fibers play important roles in supporting and maintaining human health (Prasadi and Joye, 2020). To illustrate, adequate consumption of dietary fibers is associated with lower mortality in subjects suffering from digestive, chronic, and inflammatory diseases (Aune et al., 2011; Makki et al., 2018; Reynolds et al., 2019). These findings are corroborated by studies showing an inverse correlation between dietary fiber intake and the incidence of immune-related disease (Burkitt et al., 1972; Sonnenburg and Sonnenburg, 2014). Further substantiation for the existence of functional relationships between fibers and the immune system arises from mechanistic research and intervention studies (Durholz et al., 2020; Tan et al., 2016).

Arabinoxylan is a major component of dietary fiber found in cereals and is composed of a β-(1,4)-linked xylose backbone with branches of arabinose residues substituted to the xylose O-2 and/or O-3 position. As a dietary component and as a food supplement, it has been acknowledged for its potential health-promoting qualities attributed to its immunomodulatory effects, although such a claim would not be permitted in the EU based on the available evidence (Zhang et al., 2015).

Arabinoxylans from different sources with various structures have been shown to increase the activities of the following immune cell populations *in vitro*: macrophage phagocytosis and monocyte-recruitment (Ghoneum and Matsuura, 2004; Govers et al., 2020), splenocyte proliferation (Cao et al., 2011), and natural killer (NK) cell activity (Perez-Martinez et al., 2015). Recently, we reported that preparations of arabinoxylans derived from rice and wheat could induce a non-specific memory response in innate immune cells, a process that has been termed “trained innate immunity” (Moerings et al., 2022). Considering the robust correlation between *in vitro* macrophage training and the activation of the Dectin-1 receptor by various arabinoxylan preparations (Moerings et al., 2022), we identified this receptor as the primary one involved in this immune response. In this latter study the effect of rice-derived arabinoxylans was more pronounced than that of wheat-derived arabinoxylans. Additionally, *in vivo* studies showed that oral administration of arabinoxylans promoted peripheral blood frequencies of myeloid-derived dendritic cells (Cholujova et al., 2013), NK cell and macrophage phagocytosis activity (Ogawa et al., 2005; Zhou et al., 2010), and increased seroconversion rate and antibody titers in response to influenza vaccination in elderly subjects (Laue et al., 2021), However, the uptake but also the recognition by pattern recognition receptors (PRRs) of dietary fibers can be complex, as factors such as particle size, solubility, and level and distribution of branches can be important (Goodridge et al., 2011). Despite the importance of structural characteristics, there is a lack of studies investigating the effects of these biophysical characteristics.

Dietary fibers support proper barrier function and mucosal immunity against disease development through direct and indirect interactions with gut-associated lymphoid tissue (GALT)-associated immune cells and/or epithelial cells in the small intestine (Hong et al., 2004; Li et al., 2020; Rice et al., 2005; Zheng et al., 2021). Despite numerous reports indicating different biological effects of orally administered dietary fibers, only a few have attempted to investigate their mechanisms of action and their *in vivo* bioavailability (Hong et al., 2004; Rice et al., 2005; Sandvik et al., 2007). These studies confirm the interaction of dietary fibers with intestinal immune cells and their potential to impact local immune cells in distant peripheral tissues. However, the process of intestinal passage, uptake, and interaction with immune cells, as well as intracellular trafficking, processing, and signaling, is complex. Particle size and insolubility can impact the passage through the intestinal epithelium, interaction with immune cell surface receptors, and accessibility to hydrolysis for fragment presentation or communication with other cells.

This study aims to investigate the effect of arabinoxylan particle size on innate immune training and resilience. To this end, different particle size fractions of arabinoxylan preparations from various sources were prepared and assessed for their innate immune training and resilience effects in primary human macrophages. Additionally, the effect of particle size on Dectin-1 receptor activation will be determined. Subsequently, a more physiologically relevant *in vitro* epithelial cell/immune cell co-culture model was utilized to examine the immunomodulatory effects of apical administration of arabinoxylan preparations with different particle sizes.

## 2 Materials and methods

### 2.1 Reagents

Five different arabinoxylan preparations were used in this study as previously described (Moerings et al., 2022): rice hull (RIBUS, St. Louis, MO, USA), wheat bran (Cargill, Minneapolis, MN, USA), rice bran-1 (RiFiber [RiceBran Technologies, The Woodlands, TX, United States]), rice bran-2 (Proryza® Gold [RiceBran Technologies]) and rice bran-3 (Urmatt Ltd, Wattana, Thailand). Their characteristics, including levels of LPS/LTA contamination, have been reported previously (Moerings et al., 2022).

### 2.2 Preparation of different particle size fractions

To obtain different particle sizes, the arabinoxylan preparations were first ground for 2 min in an analytic mill (IKA®-Werke GmbH, A11 basic; Staufen, Germany) and then sieved using an analytical sieve shaker at 50 Hz min^−1^ (Retsch GmbH, AS 200 control; Haan, Germany) for 2 h. The resulting particle sizes in micrometers (μm) were as follows: for rice bran-derived arabinoxylans, the average sizes were 250–106 μm, 106-90 μm, and 90-45 μm; for wheat bran, the sizes were 106–90 μm, 90-45 μm, and 45-20 μm; and for rice hull, the sizes were 90–45 μm, 45-20 μm, and less than 20 μm. The concentrations of arabinoxylan preparations and the fractions with different particle sizes are based on total weight and have not been normalized for specific compounds.

### 2.3 Solubility, monosaccharide composition, protein content and particle size distribution of arabinoxylan size fractions

High pressure size exclusion chromatography (HPSEC) was utilized to determine the solubility of the different particle size fractions from various arabinoxylan preparations. Briefly, the fractions were suspended in water (4 mg/ml) and heated to 100 °C for 10 min. The resulting samples were diluted to a concentration of 2 mg/ml and centrifuged (18,000×*g*) for 10 min at room temperature (RT). The supernatants were analyzed using an Ultimate 3000 HPLC (Dionex, Sunnyvale, CA, USA) equipped with a Shodex RI-101 refractive index detector (Showa Denko, Tokyo, Japan). Three TSK-Gel columns (Tosoh Bioscience, Tokyo, Japan) connected in series (4000–3000–2500 SuperAW; 150 × 6 mm) were used, preceded by a TSK Super AW-L guard column (35 × 4.6 mm). A volume of 10 μL of the sample was injected and eluted with 0.2 M NaNO3 at 55 °C at a flow rate of 0.6 ml/min. Pullulan molecular-mass standards ranging from 0.2 to 780 kDa (Polymer Laboratories, Palo Alto, CA, USA) were utilized for calibration.

To determine the monosaccharide composition, the different arabinoxylan particle size fractions were hydrolyzed with 1 M sulphuric acid at 100 °C for 3 h. The resulting monosaccharides were derivatized to alditol acetates and analyzed by gas chromatography using inositol as an internal standard (Englyst and Cummings, 1984). The presence of uronic acid (UA) was determined with the colorimetric m-hydroxydiphenyl assay automated on an autoanalyzer (Skalar, Breda, The Netherlands) as described previously (Thibault, 1979). The solubility of the different arabinoxylan particle size fractions was evaluated based on the areas under the HPSEC curves in comparison to polygalacturonic acid (5 mg/ml, Sigma-Aldrich, Zwijndrecht, The Netherlands), which is a highly soluble (>99%) polymer. The fractions were dissolved in water (5 mg/ml) and agitated for 3 h before being centrifuged at 15×*g* for 5 min. The resulting preparations were then subjected to HPSEC analysis.

Protein content of the arabinoxylan particle size fractions was determined in triplicate with the Dumas method using a Flash EA 1112 N Analyzer (Interscience, Breda, The Netherlands) using the nitrogen to protein conversion factor of 6.25. Cellulose (Sigma-Aldrich) served as a negative control and methionine (Sigma-Aldrich) was used as a standard.

Particle size analysis was conducted using the Mastersizer 2000 (Malvern Instruments, Worcestershire, UK) based on laser diffraction equipped with a Hydro 2000SM Dispersion Unit. The aqueous suspensions were analysed in triplicate and averaged. The refractive index and absorption index of the particles were set to 1.33 and 0.01, respectively.

### 2.4 Isolation and culture of primary human monocytes

Buffy coats from healthy donors were obtained after written informed consent (Sanquin, Nijmegen, The Netherlands). Primary human monocytes were isolated from peripheral blood mononuclear cells (PBMCs) as described previously (Moerings et al., 2021). Briefly, PBMCs isolation was performed by dilution of blood 1:1 with sterile phosphate-buffered saline (PBS) containing 2% heat-inactivated fetal bovine serum (HI-FBS) (HyClone Fetal Bovine Serum, Fisher Scientific, Loughborough, UK) and centrifugation in Ficoll-Paque plus (GE Healthcare Life Sciences) using Greiner Bio-One™ LeucoSEP™ Polypropylene Tubes. Monocytes were purified from PBMCs using positive selection with the quadroMACS system and CD14 microbeads (Miltenyi Biotec, Leiden, The Netherlands). Monocytes were frozen in FBS with 10% dimethyl sulfoxide (DMSO; Sigma-Aldrich) and stored in liquid nitrogen.

### 2.5 Training and resilience model in primary monocyte-derived human macrophages

Monocytes (500,000 cells/well) were added to 24-well tissue culture-treated plates (Corning Costar, New York, NY, USA) and incubated for 24 h at 37 °C in RPMI 1640 – Glutamax – HEPES medium (Gibco, Bleiswijk, The Netherlands) supplemented with 10% FBS (HyClone), 1% MEM non-essential amino acids (Gibco), 1% Na-pyruvate (Gibco), 1% Pen/strep (Gibco) and 50 ng/ml macrophage colony-stimulating factor (M-CSF) (R&D systems, Minneapolis, MN, USA) as described before (Moerings et al., 2021). Briefly, resilience was induced by treatment of monocytes with 10 ng/ml LPS (Sigma-Aldrich, *Escherichia coli* serotype O111:B4) for 24 h, followed by washout and five days exposure to 5 μg/ml arabinoxylan preparation in culture medium with 50 ng/ml M-CSF. Monocytes used to apply the training model were kept in culture medium for 24 h, followed by washout and five days exposure to 5 μg/ml arabinoxylan preparation in culture medium with 50 ng/ml M-CSF. Establishment of resilience or training in the resulting macrophages was determined by stimulation with 10 ng/ml LPS on day 7. After 24 h, supernatants were collected and stored at −20 °C for further analysis.

### 2.6 Human Dectin-1 reporter cell lines

The NFκB reporter cell lines HEK-Blue™ Null1-v cells, HEK-Blue™-hDectin-1a, HEK-Blue™-hDectin-1b, HEK-Blue™-hTLR4 and HEK-Blue™ Null2 (InvivoGen, Toulouse, France) were cultured and maintained in high glucose (4.5 g/l) Dulbecco's Modified Eagles Medium (DMEM; Gibco) supplemented with 10% HI-FBS (Gibco). These reporter cell lines overexpress the Secreted Embryonic Alkaline Phosphatase (SEAP) reporter gene driven by an NFκB-inducible promoter. All cell lines were cultured and propagated according to the manufacturer's protocol. Cell passage involved trypsinization with 0.05% trypsin-EDTA (Life Technologies) using a split ratio of 1:10. Cell lines (passage 9–28) were seeded at 1 × 10^6^ cells/ml in 100 μl/well in a poly-d-Lysine coated 96-well microplate (Greiner Bio-One) overnight at 37 °C and 5% CO_2_. The following day, reporter cell lines were stimulated for 24 h with different concentrations of arabinoxylan preparations (5, 10, 100 or 1000 μg/ml), as previously described (Moerings et al., 2022). Alternatively, LPS was used as a stimulus at concentrations of 10 μg/ml and 10, 1, 0.1 and 0,01 ng/ml (Teodorowicz et al., 2017) in a total volume of 200 μl/well. Medium was used as a negative control. Subsequently, cell-free volumes of 20 μl/well were transferred to a 96 well-plate (Corning Costar) containing 180 μl/well QUANTI-Blue™ Solution (InvivoGen). Following a last incubation of 2 h at 37 °C and 5% CO_2_. SEAP secretion was measured spectrophotometrically at 635 nm (TECAN, Giessen, The Netherlands).

### 2.7 Caco-2 cell culture

Caco-2 cells (American Type Culture Collection, Rockville, USA) were cultured in high glucose (4.5 g/l) DMEM (Gibco) supplemented with 10% heat inactivated FBS (Hyclone) at 37 °C and 5% CO_2_. Cells were sub-cultured weekly upon confluence 85–95% using trypsination. Caco-2 cells were used from passage 30–40 and seeded into 1.0 μm pore transparent 24-well cell culture inserts (Greiner Bio-One) at a concentration of 0.225 × 10^6^ cells/ml in DMEM (Gibco) with 10% HI-FBS (Gibco). The cells were incubated for 21 days at 37 °C and 5% CO_2_ to differentiate into small-intestinal like epithelial cells. Apical (150 μl) and basolateral (750 μl) medium was replaced three times a week and one day prior to the co-culture establishment. To monitor integrity of the Caco-2 monolayer, transepithelial electrical resistance (TEER) was measured using a MilliCell ERS (Millipore Amsterdam, The Netherlands). Caco-2 monolayers were considered of acceptable quality if TEER values were higher than 700 Ω/cm^2^.

### 2.8 Caco-2 and macrophages co-culture model

Primary monocytes were cultured on the apical side of 0.4 μm pore transparent 12-well inserts (Greiner Bio-One) at a concentration of 0.5 × 10^6^ cells/ml in RPMI 1640 – Glutamax – HEPES medium (Gibco) supplemented with 10% HI-FBS (HyClone), 1% MEM non-essential amino acids (Gibco), 1% Na-pyruvate (Gibco), 1% Pen/strep (Gibco) and 50 ng/ml M-CSF (R&D systems). After 48 h (experimental day 2), the 24-well inserts containing small intestinal-like Caco-2 monolayers differentiated for 21 days were positioned on top of 12-well inserts with primary monocytes and all media were replaced with supplemented RPMI 1640 – Glutamax – HEPES medium. To assess bioavailability and training effects of arabinoxylan preparations in this model, fresh medium (control) or 5 μg/ml arabinoxylan preparations were added to the apical side of 24-well inserts in the co-culture model and incubated for 72 h at 37 °C and 5% CO_2_. For resilience, fresh medium, 10 μg/ml LPS or a combination of 5 μg/ml arabinoxylan preparations and 10 μg/ml LPS were added to the apical side of 24-well inserts in the co-culture model and incubated for 72 h at 37 °C and 5% CO_2_. The integrity of the Caco-2 monolayer in the co-culture model was measured by TEER using a MilliCell-ERS (Millipore) apparatus before (t = 0) and after the addition of medium, 5 μg/ml arabinoxylan preparations, 10 μg/ml LPS or both arabinoxylan preparations and LPS (t = 1, 3, 6, 24, 48 and 72 h). The TEER value was normalized to the medium control and the starting time point of individual transwells. FITC-dextran 4 kDa (FD4, Sigma-Aldrich) was added to the apical compartment of the differentiated Caco-2 cells at a concentration of 250 μg/ml. After 6 h, a 100 μL sample was collected from the basolateral side, situated within the apical compartment of the macrophages, and replaced with an equal volume of fresh medium. The translocation of FD4 to the basolateral side was quantified with a spectrophotometer at excitation and emission wavelengths of 490 nm and 525 nm (TECAN). After 72 h (at experimental day 5), the 24-well inserts were removed from the co-culture model and macrophages were rested for 48 h at 37 °C and 5% CO_2_. At experimental day 7, 10 ng/ml LPS was added to the apical side of 12-well inserts containing macrophages. After 24 h, apical and basolateral medium were collected and stored at −20 °C for further analysis.

### 2.9 Cell viability assay

Cell viability of Caco-2 cells and primary macrophages in the co-culture model was determined using the MTT assay. After 72 h, following assembly and stimulation with arabinoxylan preparations, cells were washed and incubated with 500 μl medium containing 0.5 mg/ml MTT (Merck, Darmstadt, Germany) for 2 h at 37 °C and 5% CO_2_. Next, 300 μl DMSO:ethanol (1:1) was added to each well and the plate was mildly shaken for 5 min. The absorbance was measured spectrophotometrically at 570 nm (TECAN).

### 2.10 Cytokine measurements

The production of interleukin (IL)-6 and tumor necrosis factor alpha (TNF-α) in supernatants of macrophage cultures and co-cultures were determined by means of ELISA (BioLegend, San Diego, CA, USA) according to manufacturer's protocol.

### 2.11 Statistical analysis

Data from human Dectin-1 stimulation experiments were presented as the mean of three independent experiments in triplicate. All other experiments were conducted with cells from a minimum of three human donors (numbers indicated in individual Fig. legends). All data were analyzed using GraphPad Prism software version 9.0 (Graphad, La Jolla, CA, USA). Results were analyzed using one-way ANOVA followed by Dunnett's multiple comparisons test. A two-sided *P* value < 0.05 was considered as statistically significant. Data are shown as means ± standard deviation (SD).

## 3 Results

### 3.1 Particle size is an import factor in the induction of immune resilience by rice bran-derived arabinoxylan

**Fig. 1:**
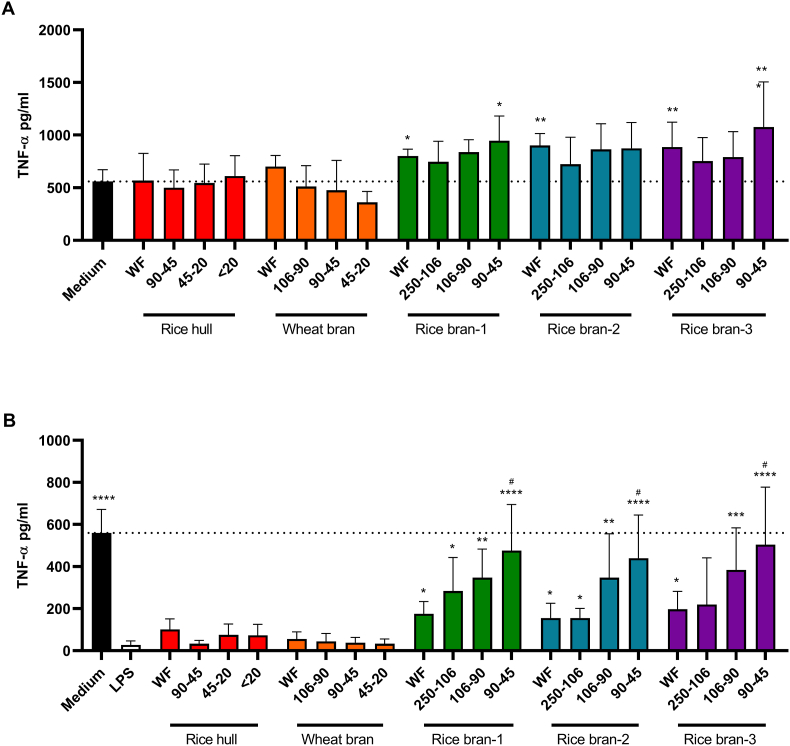
**Rice bran-derived arabinoxylan preparations induce training and resilience effects in human macrophages depending on particle size**. Production of TNF-α upon re-stimulation was evaluated in cells trained with various arabinoxylan particle size preparations (**A**). TNF-α production in LPS-primed macrophages was examined to assess induced resilience (**B**). Data are presented as mean ± SD, *n* = 6 different donors. Statistical significances are shown as: *P < 0.05, **P < 0.01, ***P < 0.001, ****P < 0.0001, compared to the no arabinoxylan (medium; **A**) or LPS (**B**) control, ^#^*P* < 0.05 compared to the WF fraction. WF, whole fiber.

In order to investigate different means and distribution of particle sizes for their potential to induce immune training or resilience effects in human macrophages, the parental rice and wheat-derived arabinoxylan preparations were grounded and sieved. Prior to stimulations of macrophages, all different arabinoxylan particle size fractions were analyzed for their solubility, monosaccharide composition, protein content and particle size distribution. Despite the poor solubility of most fractions, no changes in the other characteristics were observed between the different fractions, ruling out that different size fractions have different compositions which might impact on their immune effect (Supplementary Table 1). The particle size distributions of the fractions were different in terms of the mean particle size and corresponded to the size of the sieves used for each fraction (Supplementary Table 2). Applying the training protocol, cells were exposed to the different arabinoxylan preparations from day 2–6 followed by an LPS stimulus at day 7. Macrophages exposed to the whole fiber preparation of rice hull and wheat bran did not alter their TNF-α release when compared to control (Fig. 1A). In contrast, macrophages exposed to the whole fiber preparations of all three rice bran preparations showed a significantly (*P* < 0.05) increased release of TNF-α upon LPS exposure compared to medium control on day 7 (Fig. 1A)**.** Apart from the rice bran-1 and rice bran-3 90-45 μm fraction, the different smaller particle sizes of rice bran preparations did not result in increased levels of TNF-α compared to medium control macrophages (Fig. 1A).

Next, we investigated whether the different arabinoxylan particle size preparations could improve resilience in macrophages tolerized by an LPS trigger on day 1. Macrophages exposed to the whole fiber preparation of the three rice bran arabinoxylan preparations exhibited an increased release of TNF-α when re-stimulated with LPS at day 7 compared to the tolerance control (Fig. 1B). In line with the training results, this was not observed with the whole fiber preparation of rice hull and wheat bran. Exposing LPS-primed macrophages to the different size fractions of rice-bran arabinoxylan resulted in a size-dependent increase in TNF-α release following a secondary LPS trigger (Fig. 1B). Rice bran fractions 90-45 μm were even superior to whole fiber preparations restoring TNF-α release to medium control levels.

### 3.2 Smaller particle size fractions show enhanced potency to induce Dectin-1-mediated signaling

**Fig. 2:**
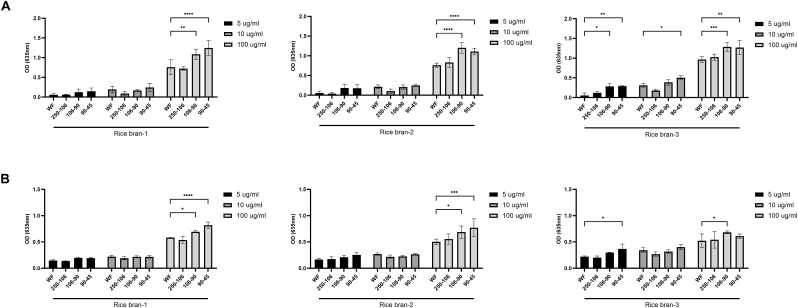
**Activation of both Dectin-1 isoforms by particle size-separated rice-derived arabinoxylan preparations**. HEK- Blue™ - Dectin-1a (**A**) and Dectin-1b (**B**) cells were stimulated with 5, 10 and 100 μg/ml of different particle size fractions of rice bran-1, rice bran-2 and rice bran-3. Data are presented as mean ± SD, *n* = 3 independent experiments and corrected for the non-transfected control cell line HEK-Blue™Null-1V. Statistical significances are shown as: *P < 0.05, **P < 0.01, ***P < 0.001, ****P < 0.0001, compared to the WF fraction. WF, whole fiber. (For interpretation of the references to color in this figure legend, the reader is referred to the Web version of this article.)

Since Dectin-1 activation is correlated with training and resilience effects (Moerings et al., 2021, 2022), we investigated whether a smaller particle size fraction also leads to an enhanced Dectin-1 activation in addition to the previously demonstrated increase in resilience effects. Both Dectin-1a (Fig. 2A) and Dectin-1b (Fig. 2B) reporter cells showed significant enhanced receptor activation compared to the whole fiber preparation at a concentration of 100 μg/ml when exposed to the 106-90 μm fractions of all rice bran preparations and to the 90-45 μm fractions of rice bran-1 and rice bran-2. Moreover, a concentration-dependent activation of both Dectin-1a and Dectin-1b was observed for all three rice bran preparations. At a concentration of 5 μg/ml, the smaller particle size fraction 90-45 μm of rice bran-3 also significantly enhanced receptor activation of both Dectin-1a (Fig. 2A) and Dectin-1b (Fig. 2B). In contrast, smaller particle size fractions of rice hull and wheat bran did not lead to an enhanced receptor activation of either Dectin-1 isoform (Supplementary Fig. 1). In further experiments, only rice bran-derived samples were used, the rice hull and wheat bran fractions were excluded.

### 3.3 Rice bran-derived arabinoxylan preparations did not affect barrier integrity and cell viability in a Caco-2/macrophage co-culture model

**Fig. 3:**
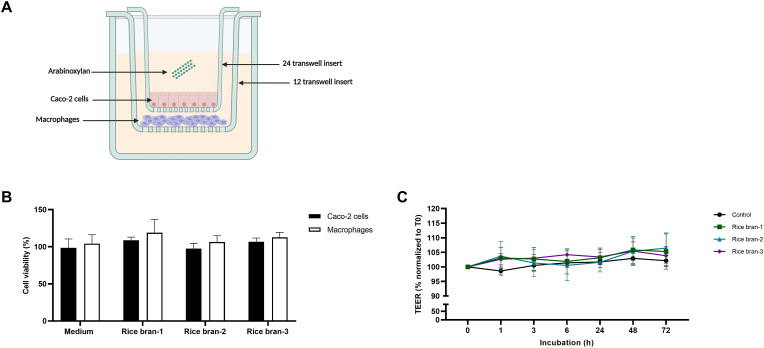
**Rice bran-derived arabinoxylan preparations had no impact on barrier integrity and cell viability in a co-culture model.** Schematic representation of the co-culture model with Caco-2 cells and primary macrophages (**A**). Caco-2 cells in the co-culture model were apically exposed to 5 μg/ml arabinoxylan preparations. Cell viability of both cell types in the co-culture model was assessed using the MTT assay after 72 h (**B**). Changes in TEER were monitored during a period of 72 h (**C**). Data are presented as mean ± SD, *n* = 3 different donors for cell viability and *n* = 6 different donors for TEER.

A Caco-2/macrophage co-culture was established by culturing human macrophages in 12-well inserts and Caco-2 cells differentiated to small-intestinal-like epithelial cells in 24-well inserts. To mimic direct interaction between the epithelium and immune cells, 24-well inserts were positioned on top of the 12-well inserts (Fig. 3A). This design allows for the cells to be in close proximity. To assess whether the assembly of the co-culture system impacted cell viability of the epithelial cells or macrophages, metabolic activity (by MTT assay) was assessed after 72 h of co-culture (Fig. 3B). In the setting with no apical stimulation (medium), no obvious decrease in cell viability could be observed measured as changes in MTT conversion. Apical exposure for 72 h with the different rice bran fractions did not reduce cell viability (Fig. 3B). To assess the effect of apical stimulation with rice bran fractions on barrier integrity, TEER was measured after 72 h of exposure. None of the rice bran fractions induced a change in TEER compared to medium (Fig. 3C).

### 3.4 Smaller rice bran-1 particle size fractions increase barrier integrity and induce innate immune training in the co-culture model

**Fig. 4:**
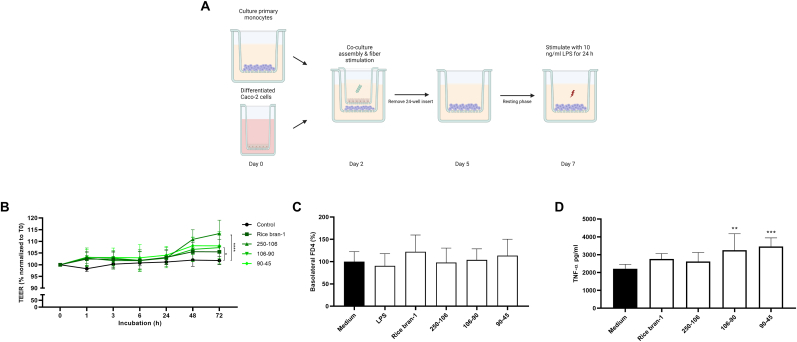
**Co-cultures exposed to smaller rice bran-1 particle size fractions display increased barrier integrity and immune training in human macrophages**. Schematic representation of the co-culture model with Caco-2 cells and primary macrophages to assess potential training effects (**A**). Co-cultures were exposed to 5 μg/ml rice bran-1 size-separated fractions. Changes in TEER were monitored during a period of 72 h (**B**). FD4 translocation was measured after 6 h by determining the accumulated fluorescent signal in the basolateral compartment (**C**). To assess the effect of particle size on the induction of innate immune training at the intestinal interface, TNF-α production was measured after re-stimulation (**D**). Data are presented as mean ± SD, *n* = 6 different donors. Statistical significances are shown as: *P < 0.05, **P < 0.01, ***P < 0.001, compared to medium control.

To investigate whether particle size plays a role in the immunomodulatory effect and sensing of arabinoxylan preparations at the intestinal interface, confluent Caco-2 monolayers were positioned on top of 12-well inserts containing macrophages and apically exposed to the different particle size fractions of rice bran-1. To assess potential training effects in macrophages, exposed Caco-2 cell inserts were removed after 3 days and macrophages were challenged with 10 ng/ml LPS at day 7 for 24 h (Fig. 4A). TEER measurements of the removed Caco-2 monolayers showed that the barrier integrity was significantly increased upon stimulation with the 250-106 and 90-45 μm fractions compared to control (Fig. 4B), while FD4 permeability was not altered (Fig. 4C). To measure if Caco-2 apical exposure to rice bran fractions induced macrophage cytokine responses, TNF-α levels were measured in the medium after removal of the Caco-2 cell inserts. None of the rice bran fractions induced a change in TNF-α release compared to medium control (Supplementary Fig. 2). Macrophage containing cell inserts were challenged with LPS at day 7, 2 days after removal of the Caco-2 inserts, and cytokine levels were measured. Compared to conditions were Caco-2 cells were unstimulated, apical stimulation with the smaller (106-90 and 90-45 μm fractions), but not the larger fractions, induced a significantly increased release of macrophage TNF-α (Fig. 4D). Furthermore, apical stimulation with the 90-45 μm fractions also resulted in a significant increase in IL-6 secretion (Supplementary Fig. 3). However, there were no significant differences observed in IL-8.

### 3.5 Reversal of LPS-induced tolerance in co-culture model macrophages by smaller particle size fractions of rice bran-1

**Fig. 5:**
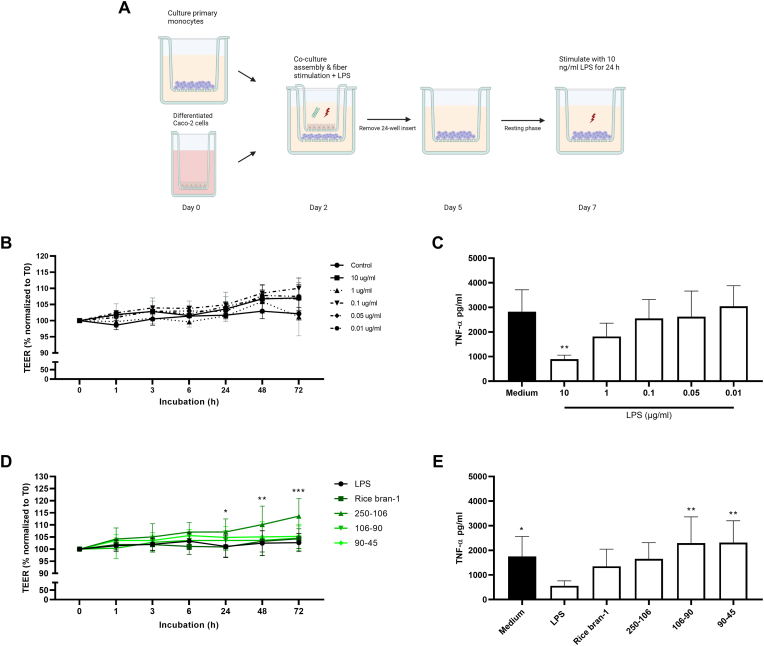
**Smaller rice bran-1 particle size fractions reverse LPS-induced immune tolerance in a co-culture model**. Schematic representation of the co-culture model with Caco-2 cells and primary macrophages to assess potential resilience effects (**A**). Co-cultures were exposed to different LPS concentrations (10, 1, 0.1, 0.05 and 0.01 μg/ml) (**B, C**) or a combination of 10 μg/ml LPS and 5 μg/ml rice bran-1 size-separated fractions (**D, E**). Changes in TEER were monitored during a period of 72 h (**B, D**). To measure LPS-induced immune tolerance, TNF-α production was measured after re-stimulation (**C**). For the effect of particle size on the induction of resilience, TNF-α was measured in supernatant of LPS-primed macrophages (**E**). Data are presented as mean ± SD, *n* = 4 different donors for LPS-induced immune tolerance and *n* = 6 different donors for resilience. Statistical significances are shown as: *P < 0.05, **P < 0.01, ***P < 0.001, compared to medium (**B**, **C**) or LPS control (**D**, **E**).

Next, the co-culture model was applied to study macrophage resilience following Caco-2 apical exposure to LPS or LPS in combination with rice bran fractions. First, we assessed the effect of Caco-2 exposure to different LPS concentrations on macrophage responses following a secondary macrophage 10 ng/ml LPS stimulation at day 7. Applying the same co-culture technique as described above, Caco-2 cells were apically exposed to increasing concentrations of LPS for 72 h, the inserts removed, and macrophages allowed to rest for an additional 2 days (Fig. 5A). At day 7, macrophages were stimulated with LPS and TNF-α levels measured after 24 h. Apical exposure to the different LPS concentrations did not affect TEER (Fig. 5B). However, Caco-2 exposure to LPS dose-dependently inhibited macrophage TNF-α release when compared to medium (Fig. 5C). To study if apical addition of rice bran-1 fractions could prevent LPS-induced macrophage tolerance, Caco-2 cells were apically exposed to 10 μg/ml LPS in combination with the different rice bran fractions. In line with the training results, TEER was significantly increased upon stimulation with the 250-106 μm fraction of rice bran-1 compared to LPS control (Fig. 5D). Furthermore, apical exposure of Caco-2 cells to LPS and the smaller size fractions of rice bran-1 (106-90 and 90-45 μm), but not the larger fractions, could rescue macrophage secondary LPS responsiveness measured as an increased release of TNF-α compared to Caco-2 exposure of LPS alone (Fig. 5E). A trend towards an increase in the production of IL-6 was observed after apical exposure with the 90-45 μm fraction of rice bran-1 (Supplementary Fig. 3). To investigate whether this prevention of LPS-induced immune tolerance was caused by neutralization of LPS by the rice bran-1 particle fractions, Toll-like receptor 4 (TLR4) activation upon combined exposure was determined in a TLR4 reporter cell line. None of the rice bran-1 fractions significantly reduced TLR4 activation following stimulations with different dosages of LPS, suggesting that the rice bran fractions have no functional impact on LPS-receptor interactions (Supplementary Fig. 4).

## 4 Discussion

Our experiments built upon our previous findings and confirmed the ability of arabinoxylans to induce resilience and training effects of the innate immune system. When specifically examining the effects of particle size, which was the main objective of this research, we demonstrated that rice bran-derived poor- and/or in-soluble arabinoxylan preparations with smaller particle sizes are able to enhance resilience effects in human macrophages, compared to compositionally similar larger sized particles. Furthermore, we demonstrated for the first time that small particle size fractions of rice bran-derived arabinoxylan preparations increase intestinal barrier integrity and induce macrophage immune training and resilience in an *in vitro* co-culture model.

It has been previously reported that the immune-modulating potential of arabinoxylans is influenced by their structural characteristics such as branching frequency, solubility, molecular weight, and particle size (Zhang et al., 2015; Izydorczyk and Dexter, 2008; Saulnier et al., 2007). The yield of the 90-45 μm fractions of rice bran was 1 w/w% of the parental preparations for each fiber, resulting in only 0.3 w/w%, 0.4 w/w%, and 0.3 w/w% of particulate carbohydrate present in the fractionated substrates. Despite the small amounts of particulate carbohydrate content, all rice bran 90-45 μm fractions were immune stimulating. This suggests that arabinoxylan must be presented in an immobilized form, similar to the fact that particulate preparations of fungal β-1,3 glucans are required for signaling (Rosch et al., 2016). In line with our previous data (Moerings et al., 2022; Han et al., 2020), our results again suggest that rice bran-derived arabinoxylan preparations, characterized by a high arabinose/xylose ratio with low solubility in medium, induce a robust induction of both innate immune training and resilience. In contrast, arabinoxylan preparations from wheat, which have a lower arabinose/xylose ratio and high solubility, did not induce immune training or resilience. Furthermore, composition analysis showed that the differences found in activity could not be explained by differences in chemical composition between larger and smaller particle size fractions. However, we have to point out that the number of particles per incubation is likely to be different for each fraction since test concentrations were calculated on a weight basis. Smaller particle size fractions did induce more Dectin-1 activation compared to larger size fractions at equal weight in our study, suggesting that smaller fractions offer relatively more Dectin-1 ligands. Smaller fractions appear more effective, but in absence of impact of the whole fiber preparation, no activity was observed even in the smaller fractions of rice hull and wheat bran (Moerings et al., 2022; Elder et al., 2017). Increased Dectin-1 activation could underlie the improved effects on macrophage training and resilience as we, and others, have already shown that Dectin-1 activation is important for the induction of these type of macrophage immune responses (Moerings et al., 2022; Goodridge et al., 2011; Mata-Martinez et al., 2022; Rosas et al., 2008).

Thus far, the majority of studies exploring the mechanisms underlying the immunomodulatory effects of dietary fiber have relied on relatively basic *in vitro* models, wherein macrophages were directly exposed. Although it has been described that macrophages continuously sample the intestinal mucosa for commensal bacteria and food- and environmental antigens (Muller et al., 2020), models comprising only this cell type possess inherent limitations. For β-glucans specifically, capture GALT-associated immune cells and/or epithelial cells has been demonstrated (Hong et al., 2004; Rice et al., 2005). Once internalized, β-glucan containing macrophages migrate to the spleen, lymph nodes, and the bone marrow where immune modulation takes place (Hong et al., 2004). Consistent with previous studies (Baggio et al., 2022; Li et al., 2022), arabinoxylan preparations showed a clear effect on barrier integrity in our study, with the smaller particle size fractions of rice bran-1 increasing TEER. Interestingly, we demonstrated that apical exposure to smaller rice bran-1 particle fractions of 106–90 and 90-45 μm in the co-culture model elicits a strong induction of both innate immune training and resilience, as measured by the increased release of TNF-α. Since these fractions did not reduce TEER or increase FD4 translocation levels, it is tempting to speculate that the macrophages were able to physically sense and interact with the rice bran-1 representations applied to the apical side of the epithelial cells. This phenomenon has already been demonstrated for human macrophages and dendritic cells co-cultured with epithelial cells (Noel et al., 2017; Rescigno et al., 2001). However, there is no direct evidence that this also applies in our co-culture model, as rice bran-1 particles may also be transported transcellularly by epithelial cells (Menard et al., 2010). Therefore, further research is needed to determine whether macrophages can sense, bind, and capture rice bran-1 particles though appendages that extend across the epithelial monolayer without disrupting the epithelial barrier.

Our findings suggest a supportive role of particle size on the immunomodulatory effects of rice bran-derived arabinoxylan preparations (*i.e.,* an increase in bioactivity leading to increased Dectin-1 activation followed by enhanced training and resilience effects). We have shown that particle size is important for the bioactivity of arabinoxylans at equal distribution by weight. Similar to β-glucans, arabinoxylans have the ability to directly stimulate the immune system and also have a strong potential to induce innate immune training and resilience (Moerings et al., 2022). The internalization of β-glucans can influence immune responses locally and systemically through the induction of trained immunity within the myeloid cell compartment in the bone marrow (Chavakis et al., 2019; Mitroulis et al., 2018). This trained immunity-related increase in myelopoiesis leads to improved response to secondary challenges and provides protection against infections. Furthermore, clinical studies have demonstrated that oral intake of dietary fibers prevent upper respiratory tract infections and support influenza vaccination efficiency (Laue et al., 2021; Zhong et al., 2021). However, administering β-glucan orally to healthy human volunteers for seven days did not lead to detectable serum β-glucan levels and did not have any effect on modulating the innate immune response (Leentjens et al., 2014). This can possibly be explained by the short-term use of oral β-glucan administration. With respect to arabinoxylans, studies have demonstrated that their oral intake can induce immunomodulatory effects in animals (Cao et al., 2011; Badr El-Din et al., 2008; Ghoneum and Abedi, 2004). Based on our results in the co-culture model, demonstrating a training effect for arabinoxylans similar to β-glucans, one could argue that arabinoxylans may act similarly to β-glucans. However, at present there is still insufficient data that substantiate the absorption of arabinoxylans or their capacity to train the innate immune system as the underlying mechanism for their systemic immune effects. Therefore, further studies are needed in the future to explore and confirm these findings.

## 5 Conclusion

In conclusion, the current study demonstrates that smaller particle size fractions of rice bran-derived arabinoxylan preparations induced enhanced trained immunity and resilience in macrophages. The higher activity of smaller particles may depend on the increase in Dectin-1 activation. Furthermore, this study shows that smaller particle size fractions also induced training and resilience effects in the established co-culture model. The explanation for this phenomenon remains unclear, but it could be due to increased bioactivity across the epithelial barrier.

## CRediT authorship contribution statement

**Bart G.J. Moerings:** Conceptualization, Formal analysis, Investigation, Visualization, Writing – original draft. **Suzanne Abbring:** Writing – review & editing. **Monic M.M. Tomassen:** Writing – review & editing. **Henk A. Schols:** Writing – review & editing. **Renger F. Witkamp:** Supervision, Writing – review & editing. **Klaske van Norren:** Writing – review & editing. **Coen Govers:** Supervision, Writing – review & editing. **Jeroen van Bergenhenegouwen:** Supervision, Writing – review & editing. **Jurriaan J. Mes:** Supervision, Writing – review & editing, All the authors have read and agreed to the published version of the manuscript.

## Declaration of competing interest

The authors declare the following financial interests/personal relationships which may be considered as potential competing interests:J.v.B. is employed by Danone Nutricia Research. The remaining authors declare that the research was conducted in the absence of any commercial or financial relationships that could be construed as a potential conflict of interest.

## Data Availability

Data will be made available on request.
