# Supplementary material for: Rice-derived arabinoxylan fibers are particle size-dependent inducers of trained immunity in a human macrophage-intestinal epithelial cell co-culture model
Source: Curr Res Food Sci. 2023 Dec 20;8:100666. doi: 10.1016/j.crfs.2023.100666 (PMC10765302; doi:10.1016/j.crfs.2023.100666)
Supplement: Multimedia component 1 [file mmc1.pdf]

Supplementary data

Supplementary Table 1. Physicochemical characteristics of different arabinoxylan particle size preparations [a].

Arabinoxylan fractions	Relative Solubility (%)	Ara/Xyl ratio	Monosaccharides (mol%)							Protein content (%)	Total saccharide content (w/w%)	Yield (w/w%)
			Rha	Ara		Man	Gal	Glc	Uronic acid			
Rice hull	2	0.17	0	5	30	2	2	59	4	2	48	45
Rice hull 90-45	1	0.12	0	4	34	0	1	58	3	2	49	40
Rice hull 45-20	2	0.12	0	5	43	0	1	47	4	3	31	4
Rice hull <20	2	0.14	0	6	44	0	0	45	4	3	28	1
Wheat bran	90	0.21	0	15	70	0	0	14	1	1	94	90
Wheat bran 106-90	90	0.24	0	16	66	0	0	17	1	1	91	29
Wheat bran 90-45	92	0.21	0	15	72	0	0	13	1	1	89	58
Wheat bran 45-20	91	0.19	0	14	72	0	0	13	1	1	90	2
Rice bran-1	7	0.89	0	16	18	2	4	53	7	20	27	34
Rice bran-1 250-106	10	0.89	0	16	18	3	3	52	7	20	36	28
Rice bran-1 106-90	24	0.93	0	13	14	3	2	61	7	20	30	6
Rice bran-1 90-45	20	0.82	0	14	17	3	3	57	7	21	27	1
Rice bran-2	9	0.81	0	13	16	1	3	62	5	20	38	41
Rice bran-2 250-106	7	0.80	0	16	20	1	3	55	6	20	41	31
Rice bran-2 106-90	18	0.86	0	12	14	1	2	66	5	20	44	9
Rice bran-2 90-45	14	0.81	0	13	16	1	2	63	5	20	38	1
Rice bran-3	2	0.89	0	24	27	1	5	33	8	17	35	36
Rice bran-3 250-106	3	0.81	0	29	36	2	5	20	8	17	37	18
Rice bran-3 106-90	4	0.89	0	24	27	1	5	34	9	22	35	17
Rice bran-3 90-45	3	0.92	0	24	26	2	5	36	8	28	31	1

Rha rhamnose, *ara* arabinose, *xyl* xylose, *man* mannose, *gal* galactose, *glc* glucose. [a] Arabinoxylan preparations listed in this table were first treated to remove LPS and subsequently analyzed. [a] Preparations listed in this table were first treated to remove LPS and subsequently analyzed.

Supplementary Table 2. Particle size distribution of arabinoxylan fractions.

	D[4,3]	Dv(10)	Dv(50)	Dv(90)	Span
	........................µm........................				
**Arabinoxylan fractions**					
Rice hull	49.5 ± 0.19	8.1 ± 0.05	40.0 ± 0.17	104.5 ± 0.35	2.4 ± 0.01
Rice hull 90-45	50.9 ± 0.21	48.4 ± 0.02	75.8 ± 0.06	89.4 ± 0.17	0.6 ± 0.04
Rice hull 45-20	36.8 ± 0.11	22.8 ± 0.12	34.8 ± 0.05	41.4 ± 0.25	0.3 ± 0.01
Rice hull <20	13.4 ± 0.08	3.7 ± 0.22	9.1 ± 0.15	17.6 ± 0.09	1.0 ± 0.01
Wheat bran	109 ± 8.95	28.3 ± 0.42	78.5 ± 2.99	198.3 ± 19.59	2.2 ± 0.16
Wheat bran 106-90	103.5 ± 2.32	91.9 ± 0.36	101.1 ± 1.12	107.1 ± 0.87	0.2 ± 0.07
Wheat bran 90-45	63.9 ± 9.31	46.5 ± 1.09	58.4 ± 2.63	86.7 ± 2.15	0.7 ± 0.22
Wheat bran 45-20	39.8 ± 5.66	21.1 ± 2.11	28.0 ± 0.87	43.4 ± 1.53	0.8 ± 0.03
Rice bran-1	241.2 ± 4.88	30.4 ± 0.51	165.1 ± 2.84	553.2 ± 11.58	3.17 ± 0.04
Rice bran-1 250-106	201.6 ± 1.62	111.1 ± 1.13	134.8 ± 3.32	243.1 ± 8.03	1.0 ± 0.04
Rice bran-1 106-90	101.1 ± 0.43	92.4 ± 0.21	98.9 ± 1.89	102.6 ± 1.52	0.1 ± 0.07
Rice bran-1 90-45	79.1 ± 2.61	51.4 ± 0.02	69.5 ± 0.97	87.6 ± 2.16	0.5 ± 0.01
Rice bran-2	248.1 ± 8.53	44.9 ± 0.45	178.8 ± 3.24	543.3 ± 21.75	2.8 ± 0.08
Rice bran-2 250-106	214.6 ± 3.51	122.9 ± 0.13	188.5 ± 1.97	226.2 ± 6.86	0.6 ± 0.09
Rice bran-2 106-90	98.7 ± 1.02	94.2 ± 0.36	102.1 ± 0.46	105.1 ± 2.27	0.1 ± 0.02
Rice bran-2 90-45	79.1 ± 2.61	51.4 ± 0.02	69.5 ± 0.97	87.6 ± 2.16	0.5 ± 0.01
Rice bran-3	306.6 ± 2.62	105.9 ± 0.47	272.2 ± 1.19	560.0 ± 7.35	1.7 ± 0.03
Rice bran-3 250-106	239.8 ± 2.34	155.8 ± 0.27	228.5 ± 1.11	244.5 ± 5.9	0.3 ± 0.01
Rice bran-3 106-90	101.0 ± 1.14	92.7 ± 0.27	99.5 ± 0.90	103.2 ± 2.56	0.1 ± 0.01
Rice bran-3 90-45	83.3± 0.15	56.1 ± 0.04	75.5 ± 0.21	86.3 ± 0.25	0.4 ± 0.02

All data are displayed in µm and represent the mean of three replicates, mean ± SD. D [4,3] = volume-weighted mean particle diameter. Dv(10) = particle size below which 10% of sample volume is found. Dv(50) = particle size below which 50% of sample volume is found. Dv(90) = particle size below which 90% of sample volume is found. Span = measurement of the width of the distribution calculated as ((Dv(90) – Dv(10)/Dv(50)).

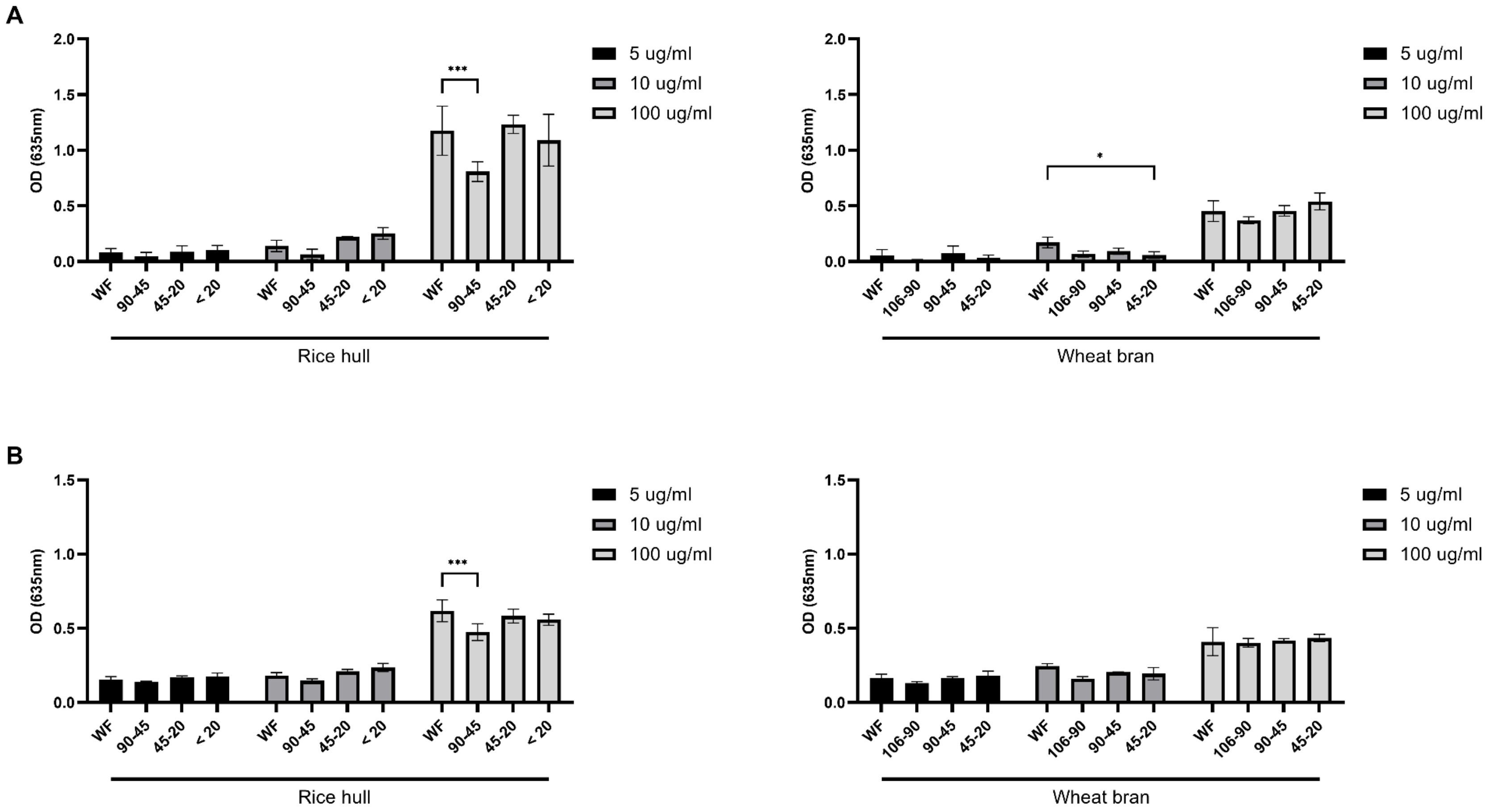

Supplementary Fig. 1. Smaller particle sizes of rice hull and wheat bran do not lead to enhanced receptor activation of both Dectin-1 isoforms. HEK-Blue™ - Dectin-1a (**A**) and HEK- Blue™ - Dectin-1b (**B**) cells were stimulated with 5, 10 and 100 µg/ml of different particle size fractions of rice hull and wheat bran. After 24 h of stimulation, the secretion of SEAP was quantified in cell-free supernatants. Data are presented as mean ± SD, *n* = 3 independent experiments and corrected for the non-transfected control cell line HEK- Blue™Null-1V. Data was analyzed using a one-way ANOVA, and statistical significances are shown as: **P* < 0.05; ****P* < 0.001. WF, whole fiber.

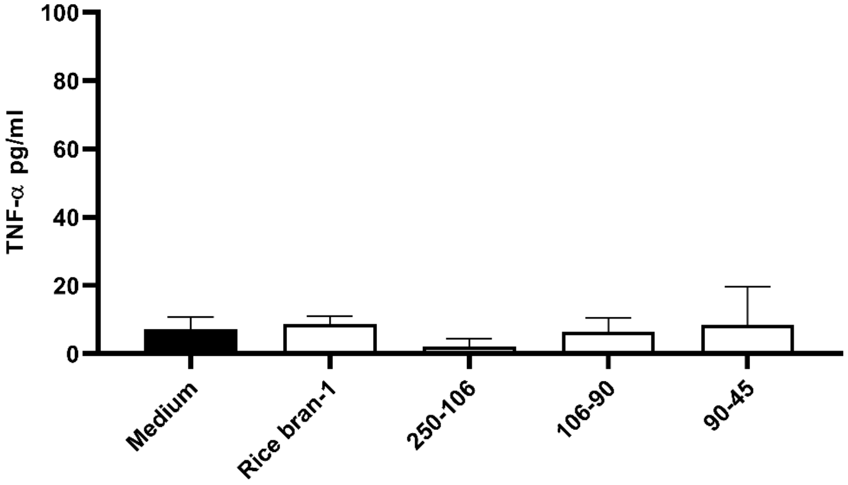

Supplementary Fig. 2. Stimulation of the Caco-2 cell inserts with rice bran-1 particle size fractions in the co-culture model does not induce cytokine release in macrophages. In the co-culture model, Caco-2 cell inserts were either stimulated with medium or 5 µg/ml of rice bran-1 size-separated fractions for 72 hours. To assess if the rice bran-1 size fractions induced cytokine release in macrophages, the TNF-α release in the apical compartment of macrophages was measured after 72 hours. Results are shown in bar graphs as averages (pg/ml ± SD) of $n = 6$ different donors. Data was analyzed using a one-way ANOVA.

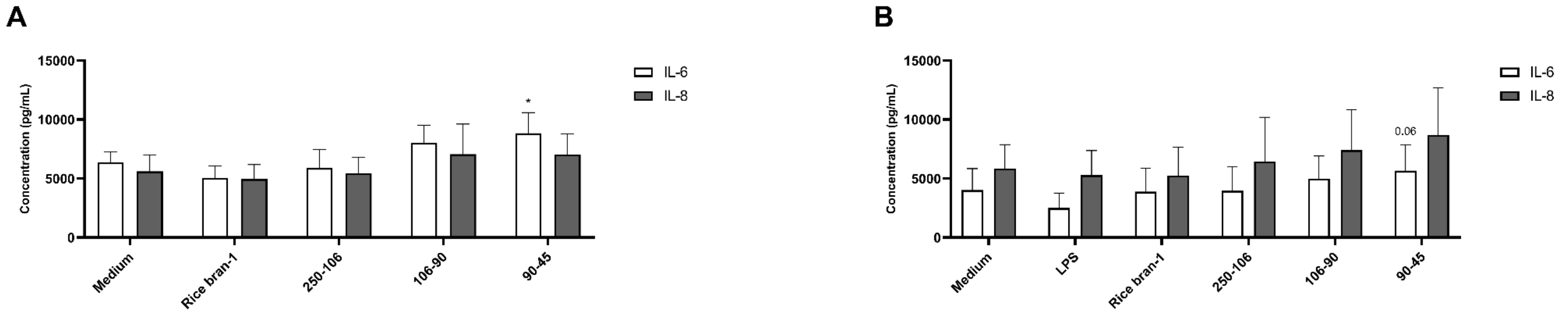

Supplementary Fig. 3. Smaller rice bran-1 particle size fractions in the co-culture result in an increased release of IL-6 and IL-8 in human macrophages. In the co-culture model, Caco-2 cell inserts were stimulated with either medium or 5 µg/ml of size-separated rice bran-1 fractions for 72 hours. The effects of these fractions on training (**A**) and resilience (**B**) were assessed by measuring the levels of IL-6 and IL-8 in the apical compartment of macrophages after re-stimulation. Results are shown in bar graphs as averages (pg/ml ± SD) of $n = 6$ different donors. Data was analyzed using a one-way ANOVA, and statistical significances are shown as: *$P < 0.05$.

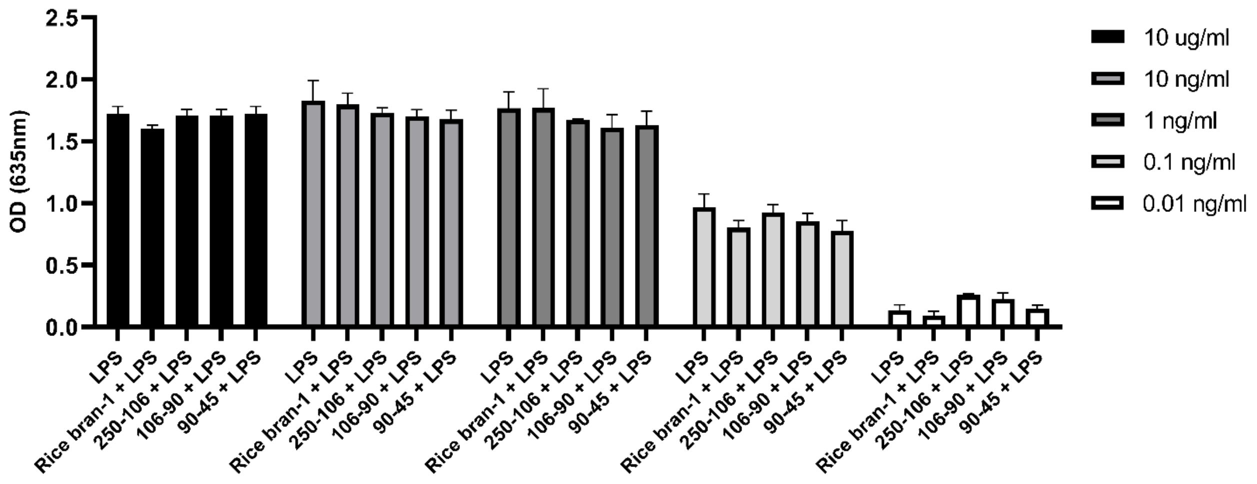

Supplementary Fig. 4. LPS-induced TLR4 activation was not decreased when combined with rice bran-1 particle size fractions. HEK- Blue™ - hTLR4 cells were stimulated with 10 µg/ml or 10, 1, 0.1 and 0.01 ng/ml LPS in combination with 5 µg/ml of different particle size fractions of rice bran-1. After 24 h of stimulation, the secretion of SEAP was quantified in cell-free supernatants. Data are presented as mean ± SD, *n* = 3 independent experiments. Data was analyzed using a one-way ANOVA followed by Dunnett's multiple comparisons test.
